# The Athlete’s Paradox: Adaptable Depression

**DOI:** 10.3390/sports10070105

**Published:** 2022-06-30

**Authors:** Weronika Jasmina Forys, Tracey Tokuhama-Espinosa

**Affiliations:** 1 Division of Continuing Education, Harvard University, 51 Brattle Street, Cambridge, MA 01238, USA; 2 Faculty of Arts and Sciences, Extension School, Harvard University, 51 Brattle Street, Cambridge, MA 01238, USA; tracey.tokuhama87@post.harvard.edu

**Keywords:** athletes, adaptable depression, adaptation, overtraining, depression, allostatic load, blunted hormone responses, hormonal conditioning of an athlete

## Abstract

We proposed that an athlete’s depressive symptoms may be different from the general population in etiology if considered from the context of a depressive disorder. By shifting focus from a limited notion of symptoms onto a comprehensive model of depression, the full scope of the phenomenon becomes clearer. This paper investigated the relationship between neurotransmitters and allostatic load to explain the incidence of depression among elite athletes. This literature review extensively analyzed exercise-induced neurohormonal imbalance resulting in depressive states among athletes. The research revealed that 5-HTTLPR polymorphism, brain-derived neurotrophic factor (BDNF), extensive psychological demands, social stigma, and overtraining syndrome (OTS) may all contribute to a unique version of depression. The research revealed that the biological standards of athletes differ from those of non-athletes, to the point that the new model may be useful, thereby introducing the new term “Adaptable Depression (AD)” to the literature. This framework suggests a new direction for future research to precisely measure the neurotransmitter-related brain changes that result in “Adaptable Depression” in athletes and to establish a better understanding of the depressive tipping point.

## 1. Introduction

Training in professional sports is an exhausting process [1] that often spans decades if one wants to achieve mastery in a specific discipline [2]. However, the length of this process is often not as strenuous to endure as an athletes’ daily training regimen [3]. Ironically, while training their bodies for peak performance, mental well-being often fails athletes at different stages of their lives and may be the cause of further health problems [4,5]. As athletic muscles and cardiovascular system adapt, so does the brain neurohormonal balance [6,7]. While some athletes find the balance between mental and physical well-being, others suffer the consequences of overcompensating for perfect physical states and compromising their mental well-being for physical superiority [8,9,10]. There is literature to support the prevalence of mental health disorders in elite athletes, as compared to the general population (Table 1). This paper explored the neural correlates of depression and overtraining to determine if the bodily exploitation that comes along with training in professional sports plays a role in cases of depression among professional athletes [11,12].

The training, which is designed to prepare the athlete for high performance competition, is an endless internal struggle for the body. The body constantly strives for homeostasis, a term used to describe the inner equilibrium of systems and physiological parameters [84]. Consequently, to endure the continually higher demands placed on the body, the organism recognizes training as a stressor and adapts [85]. This constant adaptation to stress requires energy and is referred to as allostatic load [86]. Research has suggested that professional athletes were at the same or higher risk for depression as the general population [25,26,27,28,29,33,35,87,88,89,90,91]. Moreover, it was documented that extensive athletic training could initiate a stress response in the hypothalamus–pituitary–adrenal (HPA) and sympathetic–adrenal–medullary axes [92]. Furthermore, an imbalance of the HPA axis have been connected to depression [93,94,95].

The brain is the mediating organ of the stress reaction; it determines what is stressful and coordinates multiple bodily responses to stressors [96]. A fundamental part of the brain that regulates fear responses is the amygdala. The abnormal enlargement of the amygdala explained atypically heightened fear responses, enhanced fear conditioning, and widespread chronic pain [97]. Chronic stress can contribute to alterations in the brain. In particular, it can cause amygdala hypertrophy, which, in turn, can contribute to impaired HPA axis regulation [98]. HPA dysregulation plays an important role in the onset of stress-related pathophysiology [99]. Stress-related pathophysiology may include depression [100]. This chain of relationships suggested that there could be evidence that allostatic load is related to depression [101].

The current research has indicated that it is not yet fully understood how chemical imbalances caused by athletic training could contribute to depressive states. The intent of this article was to appreciate the differences between athletes and non-athletes in terms of tipping points for depression [102]. A secondary objective was to consider the mutually influential factors that could be both catalysts as well as consequences in depressive states. While there has been evidence that the body and the brain are inextricably connected [103], there has been little research regarding whether extreme bodily exploitation could cause the brain to overcompensate in a way that disturbs general mental well-being. There appeared to be a gap in the literature [104] related to the relationship between neurotransmitters, allostatic load, and the explanation of the incidence of depression in professional athletes. This paper sought to better understand that relationship by examining the multiple variables that contribute to depression.

How does the relationship between neurotransmitters and allostatic load explain the incidence of depression in professional athletes?

## 2. Methodology

A transdisciplinary review of the research was necessary to explain the complicated relationship between depression (MDD) and overtraining syndrome (OTS) in athletes. While numerous theoretical models exist related to depression, most have emerged from a single disciplinary viewpoint. We suggested that a transdisciplinary perspective, in which studies from psychology, neuroscience, and mental/physical health were considered, offered a more complete assessment of the complexities in an athlete’s life regarding depression and overtraining syndrome.

To frame the focus on theories of depression in a historical context, a literature review was used to answer the proposed research question using peer-reviewed journals over the past 30 years. In this study, one exception was made to include a landmark study explaining the concept of homeostasis in 1932 (Cannon, 1932). A systematic search up to and including June 2022 was conducted of PubMed, PsycINFO databases, Harvard Online Library Catalogue (HOLLIS), and Google Scholar search engines. The inclusion criteria considered all studies of elite or professional athletes with comparisons to non-athletes published in English and Polish (the authors’ native languages). The exclusion included articles focused only on children or articles on acute physical exercise by non-professional athletes.

Main keyword choices included “athlete”, “depression”, “allostasis”, “allostatic load”, “depression risk factors”, “athletic risk factors”, “learning”, and “cognition”. When considering words related to “athlete”, the words “overtraining syndrome (OTS)”, “non-functional overreaching (NFOR)”, and “risk factors” were included. When considering “allostasis”, the words “allostatic load”, “stress response”, and “homeostasis” were included. When considering “depression”, the words “hypothalamus–pituitary-axis”, “sympathetic–adrenal–medullary axis”, “serotonin (5-HT)”, “serotonin transporter (5-HTT)”, “serotonin transporter polymorphism (5-HTTPLR)”, “BDNF”, and “proBDNF” were included. The authors "snowballed"the findings using the references list from Cadegiani and Kater 2017 and Petito et al., 2016.

The literature review findings divided “depression” into three subcategories: “homeostasis”, “allostasis and stress response” and “serotonin transporter polymorphism and major depressive disorder”. The second main category of “athletes” was divided into “athletes risk factors” and “overtraining syndrome (OTS) and nonfunctional overreaching (NFOR)”. The subcategory of “athletes risk factors” was further divided into the sub-subcategories of “depression and suicide”, “insomnia drug abuse”, and “anorexia”. The subcategory of “overtraining syndrome (OTS) and nonfunctional overreaching (NFOR)” was divided into “OTS and cortisol levels” and “OTS and depression”.

## 3. Subsections Relevant for the Subject

### 3.1. Depression

The Diagnostic and Statistical Manual of Mental Disorders (DSM-5) described major depressive disorder (MDD) as an illness objectively classified by the presence of five or more symptoms present for at least two weeks, alongside visible alterations in functioning [105]. Moreover, at a minimum, one of two prevalent symptoms must be present at all times: (1) depressed mood and/or (2) loss of interest or pleasure [105]. Furthermore, the APA divided depression into numerous distinct subcategories as indicated, including “disruptive mood dysregulation disorder, major depressive disorder, persistent depressive disorder (dysthymia), premenstrual dysphoric disorder, substance/medication-induced depressive disorder, depressive disorder due to another medical condition, other specified depressive disorder, unspecified depressive disorder” [106]. A book by Hancock and McKim presented two main theories regarding the development of MDD, including monoamine, and glucocorticoid theories, which researchers have emphasized as “two sides of the same coin”, rather than antagonistic theories [107]. The monoamine theory stated that depression was a result of reduced levels of activity of serotonin (5-HT) in monoamine systems. The glucocorticoid theory stated that the HPA axis’s hyperactivity, indicated by high levels of corticotropin-releasing hormone (CRH) and cortisol, was related to MDD. Furthermore, animal studies indicated that chronic, stress-induced hyperactivity of the HPA axis was due, in part, to the downregulation of glucocorticoid receptors in the hippocampus. Researchers have stressed that the sensitization of the stress system could contribute to depressive symptoms [107]. However, MDD is extremely complex, and the etiology is not yet fully known [108]. There are many variables that have to be considered when discussing MDD. One is that the monoamine hypothesis has been discredited, as it cannot explain many essential aspects of the mechanism of action of antidepressants, such as treatment-resistance or delayed onset of action [109].

### 3.2. Homeostasis

The term homeostasis was initially introduced in 1932 by Professor Walter B. Cannon. He wrote a landmark book containing information regarding the homeostasis of the body. He referred to homeostasis as the relationship between the autonomic system and the balance of physiological processes and explained that the body safeguards homeostasis for water, salt, sugar, proteins, fat, calcium, steady oxygen supply, and body temperature. Similarly, athlete bodies strive to preserve homeostasis despite the exertion that training schedules may deliver. Furthermore, Cannon was the first to propose that the body is always trying to return to homeostasis and that this was based on his observations of nature, as everything in nature seeks to return to its basic balance point [84].

#### 3.2.1. Allostasis and Stress Response

Tsigos et al. defined stress as a state of disharmony in the human body called allostasis [110]. They suggested that allostasis is counteracted by the bodily repertoire of physiological and behavioral reactions that aim to preserve/reinstate the endangered homeostasis. Another researcher discussed that the human stress response is mediated by the bodily stress system, which involves the central nervous system and peripheral organs [99]. He further showed that the central nervous system is impacted by “the hypothalamic hormones arginine vasopressin, corticotropin-releasing hormone, and pro-opiomelanocortin-derived peptides, and the locus coeruleus and autonomic norepinephrine centers in the brain stem” (p. 374), meaning that stress is the main contributor to pathological states in humans. Furthermore, another researcher recognized that a chronic surge in cortisol could have deleterious effects on the body, disturbing the balance of the HPA axis [111]. Noakes, another researcher, noted that fatigue also served as a protective system in the body [103] that allowed the organism to terminate the exercise before developing negative outcomes. Noakes proposed a visual representation of a circular pattern of fatigue that regulates behavior and the resulting feedback variations between muscle and brain [103]. Overall, these theories proposed that the brain and the heart limit the maximal muscle output through fatigue. McEwen et al. described the brain as a central organ of the stress adaptation response; the brain promotes adaptation (allostasis) and contributes to pathophysiological overuse (allostatic load) [96,98,112,113]. In summary, changes to a chronic stressor are adaptive in the short-term; however, when stress continues and changes persist, they become maladaptive. McEwen presented a visual representation of brain’s central role in allostasis. In the brain, perceived stress led to behavioral and physiologic responses. Behavioral and physiologic responses led to allostatic load, and allostasis led to adaptation. In addition, Davies discussed the topic of adaptive homeostasis, which was allostasis [86]. McEwen emphasized the physiological impairments of brain functioning under stressors [98]. He claimed that allostatic load could lead to impaired immunity, atherosclerosis, obesity, and atrophy of nerve cells in the brain, which he connected to MDD. This indicated allostasis maintained the body’s stability through change while the allostatic load was the stress-induced adaptation that could result in cumulative maladaptive changes. Human data shows that serotonin transporter variables could enhance one’s vulnerability to depressive states [107]. Some of these changes were related to the serotonin transporter that carries the neurotransmitter serotonin (5-HT) from the synaptic cleft back to the presynaptic neuron [107].

#### 3.2.2. Serotonin Transporter Polymorphism and Major Depressive Disorder

One such change in the serotonin transporter is a genetic serotonin transporter polymorphism (5-HTTPLR), which had been linked with a vulnerability to what we commonly refer to as depression, but researchers have found that the link should be categorized as a vulnerability to MDD [114]. This link was further reassessed by the systematic literature review conducted by Sharpley et al. [115]. Another pair of researchers presented the term endophenotypes, which are personal characteristics associated with disorders that correspond to specific biochemical measures [116]. Some have suggested that personal neurotransmission traits (endophenotypes) could be responsible for a given disorder, as they inform the unique way that a person’s brain interacts with what is happening in their environment and cause a neural network to fire differently [117]. Petito et al. indicated that even in a psychologically healthy population, there was a correlation between 5HTTLPR and a tendency for depression. Consequently, people who are not prone to developing depression may, in certain environments and under certain circumstances, carry a serotonin transporter polymorphism that could develop into depression in the future. In other words, the environment in which the organism exists could potentially play a significant role in the onset of depression.

Another potential risk factor for the onset of depression was established by Ancelin et al. [118], as they established that the variability in the serotonin transporter (5-HTTLPR) gene alleles could influence the risk of depression. Two alleles comprise 5-HTTLPR. Variation can occur in the length of each allele, where they can be either short (SS) pairs, long (LL) pairs, or mixed (one short, one long). The authors concluded that participants with two short alleles appeared to have a cortisol-related vulnerability to depression. Consequently, when participants carried a pair of short alleles, their past stressful events were associated with an increased risk of depression. Interestingly, another pair of researchers pointed to a smaller volume in the hippocampus and the association with a variant of 5-HTTLPR polymorphism in MDD patients [119].

Another study confirmed a relationship between the serotonin transporter gene and a predisposition toward depression. Bonnet et al. investigated variant SLC6A4 of the serotonin transporter gene. They found that this variant related to disinhibition as related to food intake [120]. Moreover, they found that those with a short allele of the promoter variant (SS), implying low expression, had 1.6 times more difficulty in controlling the amount of food eaten than those with the LL variation. In other words, there may be a genetic predisposition for some people to develop depression, based upon which variation of the serotonin transporter gene they inherited from their parents and their environment. This suggested that although some people may be prone to depressive states, other factors could influence their well-being. Restricted eating can lead to anorexia, and anorexia can tie back to depression. Therefore, the vicious circle is apparent.

### 3.3. Athletes

The definition of athletes has been under debate for many years. For this study, athletes were defined as those individuals who represent a national team in their respective sports and country, and/or those who are a collegiate athlete. Swan et al. (2015) further refined this definition by conducting a systematic review and describing definitions of elite athletes that ranged from semi-elite athletes to world-class elite athletes [121]. Athletes often strive for peak performance via increased training loads throughout their careers, and Gabbett contributed to research concluding that athletes’ performances increased with an increase in training load [122]. However, according to a study conducted by Peplonska et al., not only did the training loads play a significant role in athletes’ improvement, but so did the individual genetic variants inherited by a given athlete [123]. Peplonska et al. confirmed that genetic variants affected mental processes, and emotional regulation in the serotonergic pathway influenced the predispositions to athletic performance. Furthermore, the stress of professional athletics due to the loss of athletic identity, competitive failure, burnout, and overtraining took a toll on athletes’ mental health [90]. Hughes and Leavy further discussed that the prevalence of overtraining among elite athletes ranged from 20% to 60%; furthermore, researchers claimed that athlete burnout strongly correlated with affective disorders such as depression [90]. Another study discussed the definition of training for the body in the context of allostatic load and concluded that athletes training without load control could potentially lead to an adaptive imbalance of the nervous system resulting from chronic allostatic load [85]. Frank et al. suggested that the research on the prevalence of depression and influential psychological factors in athletes was scarce [124]. Aside from these few papers, this appears to be under-explored territory.

#### 3.3.1. Athletes and the Stigma of Mental Illness

Rice et al. conducted a systematic literature review and concluded that the athletic population was vulnerable to mental health problems, in effect debunking the misconception that elite athletes were devoid of mental health problems [87]. Interestingly, the “peak of competitive years for elite athletes tend to overlap with the peak age for the risk of the onset of mental disorders”. Furthermore, Rice et al. emphasized that athletes generally did not seek support for mental health problems due to the stigma that seeking help was a sign of weakness, and as a result, their research expressed the need for cross-discipline collaboration to help answer remaining questions. Another researcher proposed that athletes were often seen as pillars of health and wellness, which prevented the possibility of seeing this population as emotionally fragile [125]. He further emphasized that when one considers mental distress as a sign of mental weakness, this could lead to resistance to the idea of disclosing health concerns to family and medical providers.

#### 3.3.2. More Psychological Risk Factors

In their 2015 study, Wolanin et al. emphasized the presence of depressive risk factors among college athletes and stressed that athletes were not immune to depression [102]. On the contrary, this population may have some unique depressive risk factors due to loss of athletic identity, competitive failure, burnout, and overtraining [90]. Rao et al. proposed that the psychological context for athletes may be more complicated than previously thought [11]. In addition to the psychological complexities for athletes, there was a prevalence for sleep deprivation. Zanondai et al. highlighted the importance of sleep in athletes [74]; it was emphasized that competition and training schedules were associated with an increased risk of insomnia. This study emphasized that athletes’ sleep patterns were disrupted to the point that they sought sedative drugs (benzodiazepines (BZD)) in some cases to compensate for the lack of sleep. Zandonai emphasized that misuse of BZD could promote numerous mental dysfunctions (e.g., depression and attention deficits). Milano and other researchers concluded that a combination of sporting activity, factors of vulnerability toward eating disorders (ED), and the tendency to manipulate weight could result in “athletic anorexia” [126], (p. 2). Researchers presented a graphical representation of a vicious circle causing EDs as well as main sports that may lead to ED (i.e., riding, cycling, bodybuilding, swimming, volleyball, triathlon, wrestling, boxing, dance, figure skating, etc.) [126]. These sports may cause athletes to overly control their weight and body shape, resulting in calorie restrictions and eating disorders. Other researchers pointed out how overly obsessive regulatory behaviors may lead to stress-response activation. Durguerian et al. showed that when athletes restricted food intake, the bodily stress response was activated asymmetrically [127]. This suggested that obsessively controlling food intake could lead to adverse psychological and physiological bodily changes. Researchers have concluded that food limitation posed a challenging situation to any organism, including humans, by disproportionately activating the stress-response system. It suggested that food restriction could be one of the ways to disturb the homeostatic balance.

#### 3.3.3. Brain-Derived Chemical Risk Factors

Although psychological risk factors are critical to clearly understand this problem, chemical components of athletic risk factors must be presented. Extensive training has led to increased serotonin concentrations in the hypothalamus and in blood plasma concentration, in animal studies suggesting that similar consequences could result in humans [128,129]. Caperuto et al. further concluded that excessive serotonin excretion was correlated with decreased endurance performance [128]. Furthermore, two decades ago, it was discovered that athletic training led to a decline in serotonin receptors (5-HT2A) and increased prolactin (PRL) [130,131]. Another study from 2005 also found that serotonin appeared to increase to the point where symptoms of overtraining syndrome began to appear [132]. A more recent study also confirmed that physical training led to the decreased sensitivity of 5-HT receptors in animal studies, suggesting that this could also be true for humans [133]. These phenomena, when considered together, could point to a direction for further research to establish precise tipping points for harmful vs. protective regimes of exercise.

Another study by Uusitalo et al. concluded that overtrained athletes showed a “diminished number of 5-HTT in a left frontal lobe, which could be due to neuroplastic changes as a consequence of decreased serotonin production and release”, as compared to healthy non-athlete participants [134]. After two years, Uusitalo et al. conducted another study on a broader set of subjects and discussed the difference between serotonergic pathways in overtrained and healthy athletes’ brains [135]. Researchers conducted a follow-up years later to determine if anything had changed and found a symptomatic difference but no physiological change. In other words, every subject in the overtrained test group was found depressed, ranging from mild to severe [135], but no physiological change was found among test subjects. This discovery suggested that the initial differences could be due to another part of the brain involved in the serotonergic pathway.

In addition to serotonin receptors, there appeared to be another chemical risk factor–brain-derived neurotrophic factor (BDNF). This factor is a member of the neurotrophins family, supporting synaptic plasticity and improving neuronal survival [136]. BDNF is a fascinating molecule as it crosses the blood–brain barrier. Peripheral levels of BDNF have been presented to the public as potential biomarkers of mental health [137]. Interestingly, it has been assumed that the BDNF is the mediator of beneficial effects of physical activity on the brain, but the literature has reflected controversies on this topic [138]. Moreover, until 2014, “almost no study focused on the impact of long-term training on cognitive health, and serum BDNF level in well-trained athletes”, leaving a gap in literature [139]. In one of the first studies of this kind, led by Babaei, lower resting levels of serum BDNF were found in more active individuals, and the researchers hypothesized it could “reflect some kind of adaptation or downregulation of BDNF synthesis or releasing mechanisms [139]”. Other relatively new studies confirmed this supposition, suggesting that excessive athletic regime may lead to decreased basal BDNF serum levels [140,141].

Adaptation and downregulation aligned with previously discussed findings of athletic regimen’s effect on serotonin receptors. This reasoning could be plausible as the brain is highly adaptive, and BDNF and serotonin receptors belong to the same serotonergic pathway. Another study from 2014, led by Homberg, highlighted that serotonergic and brain-derived neurotrophic factor structures acted synergistically. In other words, these systems were interdependent. Furthermore, this study found that 5-HT-BDNF interactions could be relevant for etiology of mood disorders [142]. This hypothesis was validated by the study conducted in 2019, which found that that chronic corticosterone administration significantly reduced BDNF levels in rodents. The study suggested that acute stress led to the activation of the HPA axis for a short time, but afterward, the body was able to return to homeostasis. However, when the HPA axis was activated chronically (as well as glucocorticoid levels), alterations occurring in the brain could lead to depression [143]. A study from 2020 summarized that microinjections of proBDNF (precursor of BDNF) were shown to induce depressive-like behaviors in rodents. In addition, Pitsillou et al. noted that mild-stress-exposed mice had decreased BDNF levels [144]. Pro-BDNF is the precursor to the synthesis of BDNF, which has been shown to exhibit the opposite effect of mature BDNF [145,146].

### 3.4. Overtraining Syndrome (OTS) and Nonfunctional Overreaching (NFOR)

Although extensive exercise can compromise health on a chemical level, proper training-load management could help alleviate adverse symptoms. In this regard, it is essential to know that there are dichotomous training schedule approaches. Meeusen et al. [147] concluded that two forms of training schedules existed, one being beneficial and the other not. Beneficial training was named functional overreaching while the non-beneficial framework was identified as nonfunctional overreaching (NFOR). They emphasized that while NFOR was not beneficial to an athlete’s performance, there was a slight distinction between NFOR and overtraining syndrome (OTS). A keyword in recognition of OTS could be referred to as prolonged maladaptation of biological, neurochemical, and hormonal mechanisms [147,148]. Although overtraining (OT) and overreaching (OR) sound similar, they refer to different forms of decrements in performance capacity. Overtraining (OT) refers to a long-term decrement in capacity, and overreaching to short-term [149]. Meeusen et al. emphasized that repeated exposure to stress could lead to altered responsiveness to subsequent stressors [147]. Another researcher, Myrick, also emphasized the dichotomy between NFOR and OTS [150]. McEwen highlighted that acute stress intensified not only hypothalamic monoamine secretions but also corticotropin-releasing and adrenocorticotropic hormones (ATCH) that served a role in bodily stress-response function [96]. Furthermore, chronically elevated adrenal glucocorticoid secretion as a response to chronic stress could play a vital role in the desensitization of the higher brain center reactions to stressors. The endocrine system plays a central role in adaptation to chronic stress and the response to acute stressors. McEwen emphasized that a whole array of mechanisms was involved in such adaptation, acting as a cascade that triggered and led to the biological effects of the hormones [96].

#### 3.4.1. OTS and Cortisol Levels

Until the studies of Cadegiani et al., little was known about the thresholds for OTS in athletes [151,152]. OTS was diagnosed symptomatically and was subjective [12]. Cadegiani et al. demonstrated that the objectivity of the measurement of OTS was achieveable [151,152]. They suggested that there were intrinsic dysfunctions of the HPA axis response to a stress condition in OTS-affected athletes, as compared to healthy athletes. Furthermore, they established that the hypothalamus and the pituitary (not adrenals) were likely responsible for cortisol-response alterations. The first concept was that moderate-to-intense physical activity elicited hormonal conditioning that extended beyond exercise, which researchers have referred to as hormonal conditioning of the athlete [151]. The second newly emerging concept suggested that when healthy athletes were influenced by hormonal conditioning, those affected by OTS appeared to have maladaptive hormonal conditioning, as they had blunted hormone responses to stress. Researchers defined this blunting as a deconditioning process, indicating decreased performance observed during OTS. Cadegiani et al. intended that these concepts would fill the gap in understanding causal mechanisms observed in several responses to harmful situations, such as psychiatric conditions observed in athletes [151]. Since then, the authors have been developing their research, and through [153,154,155,156], they arrived at the precise thresholds for athletes where the OTS should be diagnosed [157], namely EROS-clinical, EROS-simplified, and EROS-complete. Buyse et al. pointed out that although most literature agreed that NFO and OTS were located on a linear continuum of maladaptation of the HPA axis, the hormonal levels of the HPA axis could not be used as predictors [158]. These results confirmed previous findings of Cadeigiani et al., revealing that altered responses in adrenocorticotropic hormone (ACTH), human growth hormone (GH), and prolactin (PRL) of OTS and NFO affected test subjects [151,158]. Furthermore, [111] established that the chronic dysregulation of the HPA axis was associated with the onset and course of several psychosomatic and psychiatric disorders, the most common being MDD. This phenomenon was not exclusive to athletes; however, for the clarity purposes of this work, the researchers viewed this through the athletic lens and while considering the complexity of the whole process.

#### 3.4.2. OTS and Depression

Petito et al. established a statistically significant relationship between 5-HTTLPR polymorphism, neuroticism, and sport-related stress that forecasted adverse mental health consequences (e.g., depression) in elite athletes [117]. According to these researchers, serotonin neurotransmission played a leading role in regulating the activity of the central nervous system. The serotonin transporter gene (SLC6A4) encode the serotonin transporter protein, which acts as a fundamental mechanism by removing serotonin from the synaptic cleft. Petito et al. further explained that “the promoter region of the SLC6A4 gene contains a polymorphism with short (s) and long (l) repeats in a region: 5-HTT-linked polymorphic region (5-HTTLPR)” and individuals who had either one or two duplicates of short alleles had statistically more significant measures of neuroticism than those homozygous for the long allele [117]. Moreover, neuroticism may contribute to depressive symptoms in elite athletes dealing with competitive situations, and significant correlations were evidenced between neuroticism and symptoms of anxiety and depression. Altamura et al. concluded that 5-HTTLPR was statistically significantly associated with neuroticism, the choice of stress-related coping strategy, and anxiety symptoms in athletes [159]. The choice of stress-coping strategy in athletes happened to be the “focus on and venting of emotion” (FVE) strategy, which is the tendency to focus on venting feelings of distress. This finding suggested that nature not only controlled the athletic propensity for depressive states, but nurture also contributed so that athletes could learn to cope better with emotions. Furthermore, they found that FVE and neuroticism mediated the association between the 5-HTTLPR and the symptoms of anxiety, suggesting that although several variables accounted for MDD, their combination in a specific sequence increased the likelihood of depression. Altamura et al. also reported that the growing body of evidence suggested that elite athletes could experience subsyndromal symptoms due to significant sport-related stressors (e.g., injury, overtraining) [159]. In other words, subsyndromal symptoms of depression in athletes could indicate that despite depressive symptoms, the overall scope did not meet the threshold for a formal DSM diagnosis. In the next section, we examine the literature and draw analytical conclusions through a multidisciplinary lens.

## 4. Concluding Remarks

The literature revealed four important findings that together answered the research question. First, elite athletes could adapt their bodies to a new serotonin baseline through years of extensive training schedules, which significantly differ from non-athletes [128,129]. Second, on a hormonal response spectrum, elite athletes’ reaction to prolonged stress appeared to be blunted, especially in OTS-affected individuals [151,152,153,154,155,156,157]. Third, inappropriate stress responses could lead to subsyndromal depressive states (not meeting the DSM-5 threshold) [159]. Last but not least, social stigma around depression, especially in athletic culture, could lead to under-reporting and not prioritizing this issue [87].

### 4.1. Altered Biological Standards Resulting from Allostatic Load

The literature review showed a consistent trend across all studies where athletes were recognized to as a population with different biological standards. However, this was not addressed in all mental health issues in athletes. Some health concerns have a name that implicates athleticism as the main factor, such as, athletic anorexia. However, other prevalent mental illnesses, such as depression, remain unclassified adequately to athletic needs. Although athletic depressive syndromes could differ substantially from non-athletes, usually milder than in non-athletic samples [159], they should be recognized. Furthermore, though some symptoms could be less severe, that should not imply that the depression on a linear spectrum is less severe. Referring to differences in biological standards, depression in athletes could have a different set of biological characteristics and, subsequently, a different starting point. The literature review indicated a strong correlation between depression and overtraining syndrome, suggesting that the relationship was intertwined. However, there was insufficient evidence to point out a specific causal relationship. In other words, the correlation did not account for other confounding variables that may contribute to the onset and development of either depression or overtraining syndrome.

### 4.2. Hormone-Regulated Symptomatic Difference (Neurotransmitter Balance)

The literature supported that a difference existed between the hormone- and neurotransmitter-regulated depressive symptoms. In other words, athletes do not suffer the same depressive symptoms as non-athletes, as their neurohormonal balance is entirely different from non-athletes. Some researchers have referred to athletes’ depressive symptoms as subsyndromal, as they did not meet the DSM-5 threshold [159].

Serotonin may be involved in this process as athletes over the course of their career develop abnormally high levels of serotonin [128,129], which is why their brains downregulate serotonin receptors to maintain balance [133,134]. In a study conducted in 2006 on neurotransmission in serotonin reuptake in OTS and healthy athletes, neurotransmission did not show any change between the two groups, and all of the tested subjects in the OTS group were depressed [135]. Therefore, every participant in the study that was overtrained had depression [135]. This finding was particularly important as it suggested that while OTS athlete’s neurotransmission did not differ from a healthy athlete, they suffered from depression due to their blunted hormonal response preventing them from getting enough serotonin to activate the downregulated receptors [151]. Interestingly, healthy participants did produce enough serotonin to activate their receptors. In overtrained athletes, the downregulation of the receptors did not change for up to one year [135], which indicated that if they experienced a disruption in their training schedule and did not constantly deliver vast amounts of exercise-induced serotonin along with downregulated serotonin receptors, they could suffer from medical depression.

Research has shown that BDNF was enhanced in acute exercise of varying intensity, where competitive subjects were excluded from the sample [160]. Furthermore, studies have shown that excessive training periods decreased the initial value of BDNF [139,140]. A 2016 study also reassessed this assumption and found that the base level of BDNF factor in already well-trained adolescents was lower than that in the control group, which consisted of non-athletes [141]. Only prolonged training periods and pro-athlete-like regimes appeared to affect BDNF negatively. In conclusion, non-athletic training regimes appeared to improve BDNF levels, but excessive athletic training could lead to impaired cognitive performance by decreasing BDNF levels and subsequently contributing to depressive states.

### 4.3. Well-Being of Athletes

Athletes are uniquely vulnerable due to particular risk factors, such as injury, involuntary career termination, and performance pressure (Rice et al., 2016). However, a rising concern is that athletes could be under-reporting depression symptoms to present themselves as better than they are [87]. Some concerns have been noticed by coaches concerning athletes reporting depressive conditions [102]. Athletes tend not to disclose their mental health problems, as it is seen as a sign of weakness [125]. The pressure of living up to the image of being a pillar of health and wellness pushes athletes to the edges of their capabilities, and this has resulted in adverse effects, including anorexia nervosa, insomnia, and drug abuse [125]. Some have suggested that under-reporting of depressive symptoms could be embedded in the culture of athletics, where confidence is a desired trait, which could lead to overconfidence and not disclosing the lack of this trait [102]. At the same time, the pressure to be an athlete casts a shadow over the chore of taking care of one’s mind. In other words, enhanced by the athletic cultural stigma, athletes are under-reporting depressive symptoms.

### 4.4. Diagnosis

**Figure 1:**
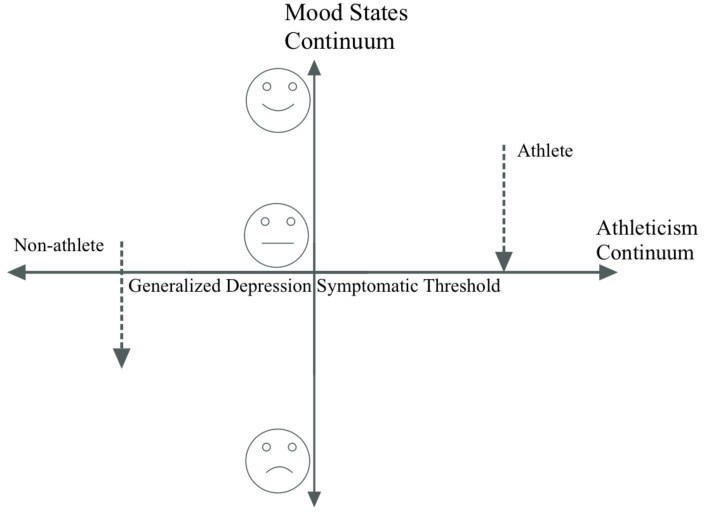
Adaptable depression in the context of mood states vs. athleticism continuum; the X-axis represents the Diagnostic Statistical Manual threshold for classifying major depressive disorder based on symptoms. The Y-axis shows a linear scale of depressive symptoms, ranging from happy to sad. Two arrows at each side of the Y-axis represent the progressive increase in depressive symptoms in elite athletes vs. non-athlete. The only difference lies in the relative starting points of non-athletes vs. athletes.

The literature revealed four significant findings, which are presented in the Figure 1.

These four findings suggested that this could be a new version of depression, which we have labeled “adaptable depression”. The name comes from adaptive processes the body of an athlete has had to undergo to achieve the initial higher point of serotonin excretion. The literature review showed that separating elite athletes from non-athletes was a constant, not a variable; therefore, addressing this distinction in every context was only appropriate as it could fill the gap in the literature. While the evidence for adaptable depression has existed for several years, most specialists only considered the evidence in their fields, thus overlooking the connection between fields. A multidisciplinary approach could provide a better understanding of depression in the context of neuroscience, psychology, human physiology, and sports medicine. This study expanded the research of experts in the field whose concepts have, to date, been considered the standard view. The literature supported the idea that being an elite athlete was a risk factor for adaptable depression and explained why the symptoms were milder than the general population. Research also suggested that elite athletes could be in a greater danger of poor mental health, partly due to demands placed on them by their sport of choice. Despite the surge in active research in recent years, there are few research papers pinpointing the underlying cause of this phenomenon. In a recent study led by McLoughlin regarding this topic, they established quantitatively that no athletes met the criteria for both severe depression and anxiety. However, “1.3%, 2.6%, 3.8%, and 24% of participants met the criteria for severe, moderately severe, moderate, and mild depression, respectively. In addition, 1.2%, 3.8%, and 21.8% of participants met the criteria for severe, moderate, and mild anxiety, respectively. No participants met the criteria for severe depression and anxiety” [30]. This finding suggested that, in line with adaptable depression theory, fewer athletes have severe depressive symptoms due to the lack of a proper symptomatic assessment of depression.

### 4.5. Answer to the Research Question

The answer to the research question “How does the relationship between neurotransmitters and allostatic load explain the incidence of depression in professional athletes?” was that the allostatic load changes the athletic serotonergic balance, resulting in milder symptoms of depression, yet, on a linear spectrum, the depression remained constant. Although the answer to this question was an expected finding, generating the concept of adaptable depression was unexpected. While additional research on distinct differences in serotonergic pathways between athlete vs. non-athlete is ongoing, adaptable depression as a concept also hinges on the concept of threshold theory. Therefore, we proposed that upon further investigation, adaptable depression may be considered a new direction for future research and possibly a new theory of depression, where adaptable depression would be categorized via relative changes in the athletic brain, rather than meeting the symptomatic threshold; the precise thresholds for OTS-affected individuals are very important for the recognition of adaptable depression. While AD is the change or adaptation, OTS-affected individuals are the maladaptive factor, but they have not yet reached burnout stage. It would appear that DSM-5-classifiable symptoms of MDD appeared at a burnout stage for athletes, whereas AD could appear when the OTS appears. The causal direction was not evident, and we hope it warrants further research and recognition. We were intrigued to realize that a new field of research may arise to answer or reject the hypothesis of adaptable depression. The relativity of adaptable depression suggested that we have two distinct relative starting points. We suggest that rather than looking for symptoms to meet a threshold, we should consider the difference from the starting point for further understanding. A novel approach to regular depression through the lens of AD honors the concept of human variability. Since no single human being is identical, the measurements used for each of the subcategories of depression could be relative to the initial points of unique individuals. What remains unanswered is what precise measurements could be used to provide scientific evidence for adaptable depression.

The dichotomy between chronic depression and adaptable depression may be subtle, but it is visible to the point that it should be recognized. The main difference, though, does not lie in symptomatic differences but the starting points of those symptoms. Only the relative change between those starting points should be recognized as adaptable depression. Simultaneously, acknowledging the existence of adaptable depression, many athletes whose learning capabilities and cognitive skills have been affected by it could seek help.

In addition, athletic coaches could provide better mental care for their athletes. Consequently, athletes’ academic performance could improve upon recognizing the first signs of adaptable depression and seeking appropriate help. Subsequently, their aspirations of attending high-level schools could have a better chance of becoming a reality. These findings have important implications within the realm of the athletic community as well as outside of it.

Individuals struggling with depressive symptoms will more frequently seek care to improve their cognitive skills if their sports idols exemplify this behavior. From the standpoint of the athletes, recognizing adaptable depression could better protect the athletic community’s health. Many athletes do not recognize that their mild symptoms may indeed reveal depression; therefore, neither they nor their coaches seek medical assistance. If mild symptoms in athletic populations could indicate severe depression, perhaps many athletes will no longer ignore their symptoms and, instead, seek professional help. Today, symptoms such as insomnia or drug abuse are considered relatively normal on the global scale of athletic performance.

### 4.6. Limitations of the Study and Recommendations for Future Studies

The main limitation of the proposed concept of AD and its effects on learning abilities was that it was based solely on a literature review as methodology. We recognize that many precise measurements must be executed to provide scientific evidence to support this newly emerged hypothesis. Therefore, we suggest that cross-disciplinary studies should be conducted to accept or reject the hypothesis. It is recommended for future studies to consider the concept of adaptable depression as a null hypothesis to provide better evidence for its existence.

The concept of adaptable depression also raised many additional questions that were impossible to answer within the scope of this study. First, the research generated questions regarding athletic overtraining syndrome that were recently answered by [157]. While the precise thresholds for OTS were discovered [157], we have yet to discover thresholds for MDD. Second, we could aim for a large-scale comparison of both MDD/burn-out and AD/OTS. This is a significant direction for future research, yet it is also a very important weak point of adaptable depression as a precise measurement to determine its existence. Therefore, further research is needed to establish the threshold of the relative difference, that is, when the change in mood and behavior is too great to be considered normal. Third, another potential area for future studies revolves around the treatment of AD. Namely, should AD be treated equally as MDD with antidepressant medications, or to a lesser extent?

Contrary to this model some research has suggested that athletic population, although vulnerable, appeared to be protected from depressive states [53]. Nonetheless, this protection may be only an illusion since scientists have not yet found evidence suggesting the brains of the athletic population (stimulated by years of training) and the general population are similar. Therefore, one of the crucial differences in depressive states between average and athletic populations could be different starting points and symptoms.

## 5. Conclusions

This paper investigated the relationship between neurotransmitters and allostatic load to explain the incidence of depression among elite athletes and how their learning abilities were affected. We analyzed exercise-induced neurohormonal imbalance that resulted in depression. However, after reviewing the literature, we found one apparent trend, suggesting that although athletes did not exhibit the same symptoms, they suffered from depression to the same extent as non-athletes due to their neurohormonal starting points being different. In other words, initial serotonin levels in athletes were higher; therefore, their depressive symptoms did not meet the *DSM-5* diagnostic criteria for depression. To protect the athletic community, it is hoped that the concept of adaptable depression will be considered worthy of further research as a potential candidate to account for the differences between elite athletes and non-athletes, as related to depressive states. 

## Figures and Tables

**Table 1:** Summary list of studies showing data referring to prevalence of mental disorder among elite athletes vs. non-athletes.

Mental Health Disorders	Elite Athletes vs. Non-Athletes
Anxiety	[13,14,15,16,17,18,19]
Bipolar disorder	[20,21,22,23,24]
Depression	[25,26,27,28,29,30,31,32,33,34,35]
Eating Disorders	[36,37,38,39,40,41,42,43,44,45,46,47,48]
Loneliness	[49,50,51,52]
Obsessive–Compulsive Disorders	[53,54,55]
Self-esteem	[17,56,57,58,59]
Self-harm	[60,61,62]
Sleep Problems	[63,64,65,66,67,68,69,70,71,72,73,74]
Stress	[70,75,76,77,78]
Suicidal feelings	[11,79,80,81,82,83]

## Data Availability

Not applicable.
